# Effects of Presenteeism on Burnout among Rehabilitation Medical Workers in Korea: Multiple Mediating Effects of Organization and Supervisor Supports

**DOI:** 10.3390/healthcare12050559

**Published:** 2024-02-28

**Authors:** Chiang-Soon Song, Sung-Ryong Ma, Jae-Young Lee, Byung-Yoon Chun

**Affiliations:** 1 Department of Occupational Therapy, Chosun University, Gwangju 61452, Republic of Korea; grsong@chosun.ac.kr; 2 Saeam Welfare Foundation, Goksung 57506, Republic of Korea; recgo2002@hanmail.net; 3 Department of Accounting & Tax, Gwangju University, Gwangju 61743, Republic of Korea; overcome@gwangju.ac.kr

**Keywords:** presenteeism, burnout, organizational support, supervisory support, rehabilitation medical workers

## Abstract

Purposes: This study aimed to examine the discriminatory impacts of two major impairment factors—job presenteeism and attention presenteeism (JP and AP)—in presenteeism on burnout and to verify the multiple mediating effects of organizational and supervisory support in their causal relationship to provide theoretical and practical implications for alleviating burnout among rehabilitation medical workers (RMWs). Methods: Participants were convenience sampled from 23 hospitals and rehabilitation medical institutions in Korea, and 494 datasets were analyzed using the R packages R-studio, Jamovi, and JASP. Results: The significant effects of JP and AP on burnout were investigated; AP (0.609) had a much higher effect than JP (0.170) on burnout among RMWs. Moreover, the multiple mediating effects of organizational support and supervisory support were verified in the JP–AP relationship and burnout among RMWs. Additionally, the absolute effect on burnout was more from AP than JP, and organizational support had a far more significant effect than supervisory support in the process of affecting burnout. Conclusions: The present study contributes to the literature on burnout by examining the relationships between presenteeism and burnout and by extending the current understanding of burnout and presenteeism to RMWs. And it is practically important to understand that the effect of AP was greater than that of JP between the two key sub-factors of presenteeism affecting burnout among RMWs, and Korean RMWs are more affected by support from the organization system than by personal support from their boss. Related theoretical and practical implications are further elaborated.

## 1. Introduction

Healthcare workers caring for COVID-19 patients in medical facilities are at the highest risk of virus transmission and infection [1]. COVID-19 requires doctors and nurses to make continuous use of personal protective equipment, aggravating physical and attentional fatigue and leading to stress and strain [2]. It also produces negative effects on both physical and mental health [3]. Therefore, they are exposed to not only the highest level of job-related risk but also a considerable amount of psychological pressure in relation to their jobs [4]. Studies have shown that the outbreak of the pandemic has led to high levels of panic, anxiety, and various psychological problems, including burnout [5], and nearly half of healthcare workers experienced burnout during the pandemic [6].

Studies on burnout among healthcare workers have reported job-related exhaustion symptoms, such as fatigue, stress, long-term isolation, anxiety, insomnia, and depression [7,8,9,10]. Therefore, defining burnout is difficult owing to the multiplicity of symptoms and side effects. However, generally, it refers to physical and mental fatigue and psychological frustration, is intimately related to one’s profession, and involves a person’s relationship with their work [11]. Maslach suggests that burnout is a syndrome of emotional exhaustion, cynicism, and reduced personal accomplishment in the process of daily contact with clients [12]. Particularly, burnout among healthcare workers negatively affects patients, organizations, and the workers themselves and is a major cause of poor quality medical services, increased medical accident rates, and suicide among medical personnel [13].

Concurrent with the high prevalence of burnout, there have been extensive studies on presenteeism among care and educational professionals such as physicians, nurses, and teachers [14]. Presenteeism is a phenomenon in which employees cannot exercise their abilities owing to illnesses or other mental and physical issues. Being forced to work in a sick state negatively impacts personal and organizational productivity [14,15,16]. Previous studies have shown that presenteeism is also prevalent among doctors and nurses, who must comply with appointments to provide medical treatment and rehabilitation services to patients.

Presenteeism can be seen as an active and risk-taking strategy [17], depicted by maximizing effort to overcome work-related demands. Therefore, based on the transactional theory of stress [18], employees may continue to work while ill, owing to a sense of job insecurity, fear of social criticism, or loyalty toward their job, colleagues, or clients.

The job demands–resources (JD-R) model states that when job demands are high and job resources are low, the risk of burnout is higher [19]. Job demands are organizational, social, or physical aspects of the job that require sustained physical and/or psychological effort from the employee, according to which, presenteeism may constitute a demand that can have an effect on employees’ health and well-being [20].

A study by the British insurer “Aviva” identified burnout among medical professionals as a result of rising presenteeism during COVID-19 [21], revealing an increasing risk of burnout because the proportion of unwell employees taking “zero” sick leave in the UK increased from 67% (before the pandemic) to 84%. This study revealed a relationship between presenteeism and burnout, which are emerging issues related to medical and rehabilitation medical workers’ (RMWs’) health. Several recent studies have demonstrated a strong correlation between these two factors [22,23,24].

However, scant attention has been paid to examining the association between burnout and presenteeism among RMWs. Moreover, related studies on burnout and presenteeism have mostly focused on particular groups, doctors and nurses, marginalizing RMWs [6,23,25,26]. Typical Korean RMWs include occupational therapists, physical therapists, and social welfare workers, excluding doctors and nurses (refer to Article 85 under Medical Service Act in Korea). Thus, we selected occupational and physical therapists as participants for this study.

Presenteeism is related to burnout among medical workers [22,26]. When employees feel unwell, their performance at work is under threat [27]. In order to reach the desired performance standards, they are to invest more effort in order to perform as well as healthy employees and not to stay sick at home. Adopting the conservation of resources (COR) theory [28], in this way, they can try to minimize their resource losses, but sickness presence impairs physical and psychological recuperation and recovery after strain or disease [29]. Therefore, our basic assumption is that the pressure to attend work while employees feel sick will give rise to feelings of burnout due to inadequate recovery [30].

Nevertheless, only a handful of studies have examined its consequences, and virtually no study has examined the discriminatory impact of presenteeism on burnout. Therefore, as we assumed that RMWs’ presenteeism was also related to burnout, we attempted to verify their causal relationship, particularly the effect of presenteeism on burnout, based on two significant impairment sub-factors that led to work loss attributed to sickness presence: “completing work” and “avoiding distraction” [31]. The former includes the degree of impairment accrued from such “job” aspects as the ability to focus on achieving goals and problem-solving, feeling energetic, and being able to work with others on shared tasks, whereas the latter includes the degree of impairment occurring from such “attention” perspectives as handling stress, feeling hopeless about finishing work, and requiring breaks from work. Thus, we operationalize the former as job presenteeism (JP) and the latter as attention presenteeism (AP).

Furthermore, studies suggest that support or encouragement from organizations or supervisors reduces burnout symptoms that negatively affect oneself, patients, and organizations [32,33,34]. According to the JD-R model, job resources may buffer the impact of job demands on job strain, including burnout [35]. A high-quality relationship with one’s supervisor and organization may alleviate the influence of job demands (work overload, emotional and physical demands) [36]. Based on the aforementioned arguments, we selected organizational support and supervisory support as variables expected to alleviate the negative impact of presenteeism on burnout among RMWs and examined their multiple mediating effects. Supervisory support refers to employees’ perception of their superior’s attention and support toward them during their work performance [37]. In previous studies, supervisory support was found to be a significant variable that reduces burnout [38,39,40]. Organizational support refers to employees’ perceptions of the extent to which the organization values their contributions and cares about their well-being [41,42]. Many researchers, like Cropanzano et al. [43], who studied the relationship between organizational support and burnout, argued that the higher the perceived organizational support of organizational members, the lower their job-related burnout [44].

**Figure 1:**
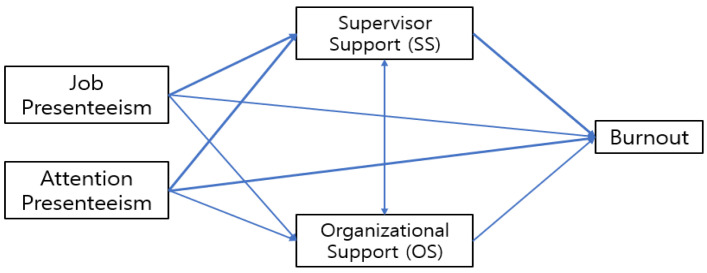
Research model.

Thus, we formulated the following two objectives of this study and designed a proposed research model (see Figure 1) as below:

1. To examine the discriminatory impacts of the two major impairment sub-factors in presenteeism (JP and AP) on burnout in the areas of work life among RMWs.
2. To verify the multiple mediating effects and their differential roles of organizational support and supervisory support as variables suggested for mitigating burnout among RMWs.

## 2. Materials and Methods

### 2.1. Study Design and Data Collection

We used a structured questionnaire to conduct a cross-sectional survey among Korean occupational and physical therapists and examined the relationships among JP, AP, supervisory support, organizational support, and burnout. Twenty-three public or private hospitals and rehabilitation medical institutions in Korea were convenience sampled. Participants were randomly sampled with inclusion criteria from Gwangju city, Chon-nam province, and Seoul metropolitan area. The inclusion criteria for the study were as follows: (1) being a rehabilitation medical worker; (2) being a relevant license holder; (3) having at least six months of work experience; and (4) voluntarily agreeing to participate (willingness to sign a written consent form). We visited the participants in Gwangju city and Chon-nam province, but for remote locations, we called them and mailed the questionnaire and consent form. We obtained consent from 260 and 245 occupational and physical therapists, respectively. Approval to conduct the study was obtained from the IRB Review Board of Gwangju University in the Republic of Korea (1906-HR-004-01). We used 494 copies for analysis, excluding 11 deemed worthless.

### 2.2. Measures

We adapted measurement scales from previous studies with satisfactory validity and reliability. All measures initially developed in English were translated and back-translated as recommended by Brislin [45]. This structured questionnaire comprised the general characteristics of the participants, a dependent variable (burnout), an independent variable (presenteeism, including the two key sub-factors), and two mediating variables (organizational support and supervisory support).

All variables, excluding demographic items, were rated on Likert 5-point scale ranging from 1 to 5 (“strongly disagree” to “strongly agree”).

#### 2.2.1. Burnout

To assess burnout, we used the Maslach Burnout Inventory (MBI), initially developed by Maslach and Jackson [46]. The MBI scale comprises three characteristics: “emotional exhaustion”, which indicates a feeling of being emotionally overextended and exhausted by one’s work; “depersonalization”, which implies a cynical and unfeeling response toward recipients of one’s care or service; and “reduced personal accomplishment”, which implies a loss or decline in confidence in the feelings of competence and successful achievement in the job. This scale comprises 16 items, with higher scores indicating more severe burnout.

#### 2.2.2. Presenteeism

For the independent variable of this study, we used 10 items from the Stanford Presenteeism Scale developed by Turpin et al. [31]. This scale represents that the higher the score, the higher the work impairment due to the presence of sickness at work. This study classified and marked JP and AP for impairment from “completing work” and “avoiding distraction”, respectively, and analyzed five items each.

#### 2.2.3. Organizational and Supervisory Support

For OS, we used four items in order of the highest factor loading indicators presented in Eisenberger et al.’s shortened version of the Survey of Perceived Organizational Support [41]. The higher the score, the higher the OS.

And we used four items for SS developed by Yoon and Lim [47]. The four items indicate the degree to which positive support can be received from the supervisors of an organization or department. Higher scores indicate a higher degree of SS.

### 2.3. Statistical Analysis

The following steps were conducted using analysis tools R. 3.6.0 packages program, R-studio, Jamovi, and JASP to analyze and visualize the results. First, the differences in the degree of burnout based on the participants’ general characteristics were analyzed using an independent sample *t*-test and analysis of variance (ANOVA) using the Jamovi program. Second, the model fit was tested by removing items with low loading values using confirmatory factor analysis (CFA). Third, to verify the effect of the independent variable on the dependent variable, we used the R package lavaan. Fourth, we examined the multiple mediating effects of OS and SS on the relationship between presenteeism and burnout using the bootstrapping technique in the JASP program.

## 3. Results

### 3.1. Socio-Demographical Differences on Burnout

To determine differences in the dependent variable burnout based on the participants’ general characteristics, an independent sample *t*-test and one-way ANOVA were conducted. Table 1 presents the study results.

The results of the *t*-test for the difference in burnout based on participants’ sex showed a significant difference. The mean burnout score of female RMWs (3.42) was significantly higher than that of males (3.02).

A one-way analysis of variance (ANOVA) (Welch’s test), which was conducted to identify differences based on age and educational background, showed that the lower the age and educational background, the higher the level of burnout, although these differences were not statistically significant. The differences in burnout based on job classification (occupational and physical therapists), double income, and the presence or absence of preschool children were also not statistically significant (*p* < 0.05). However, physical therapists (3.33) had a slightly higher burnout score than occupational therapists (3.26), and both working couples (3.21) and parents of preschoolers (3.11) showed lower burnout scores.

Regarding marital status, unmarried participants (3.35) showed a significantly higher burnout score than married participants (3.14). Accordingly, our preconceived notion that unmarried people would be more stable in their work, family, and social lives could be mistaken because people usually believe that unmarried people would have relatively more liberty of leisure time and a lower burden on work and family balance compared with married people. Therefore, it could be interpreted that married individuals would show good management of balancing work, family, and social lives, and unmarried individuals, compared to married ones, would be relatively less stable in their individual, professional, organizational, and social lives, including the potential burden of marriage. This could be the subject of future research.

Finally, the results of a one-way ANOVA to ascertain the differences in burnout based on workplace type proved that there was a statistically significant difference. Specifically, the burnout level was highest at private rehabilitation hospitals (3.47), followed by nursing (3.40), public rehabilitation (3.28), and general hospitals (3.12).

### 3.2. Validity and Reliability

We used the R and JASP packages to analyze the model fit of the structural equation model. To ensure that all variables were distinct constructs, we performed a CFA and removed one and two items from JP and AP, respectively, as their factor loading indicators were lower than 0.4, as recommended by Stevens [48].

The commonly used χ^2^ test (chi-square test) had a result of 212.228 (*p* < 0.01), which is not model-compliant but can be ignored if the sample size exceeds 200 and other indicators are acceptable [49]. Therefore, we determined model fit by comprehensively examining the other fit indices.

In this study model, the RMSEA representing the mean difference of covariance residuals was 0.059, the RMR was 0.051, and the SRMR was 0.046, which were found to be suitable as they were all less than 0.08 [50,51]. Then, as CFI, NFI, TLI, IFI, and GFI were all higher than 0.9 [52,53], the model fit was confirmed (presented in Table 2).

We also verified the average variance extracted (AVE) values and Cronbach’s alpha to verify the convergent and discriminant validity and the internal consistency reliability of the potential factors of the structural model (Table 3).

As the AVE value is the size of the variance explaining the latent factor, if it is 0.50 or more, it is judged that there exists convergent and discriminant validity [54]. Additionally, if Cronbach’s α value is greater than or equal to 0.6, the internal consistency reliability of all the scales is proven [55]. In this study, all AVE indices of the latent factors were >0.50, and Cronbach’s alpha values were over 0.75. Consequently, we were confident in the validity and reliability of each latent factor.

### 3.3. Impact of Presenteeism on Burnout

**Figure 2:**
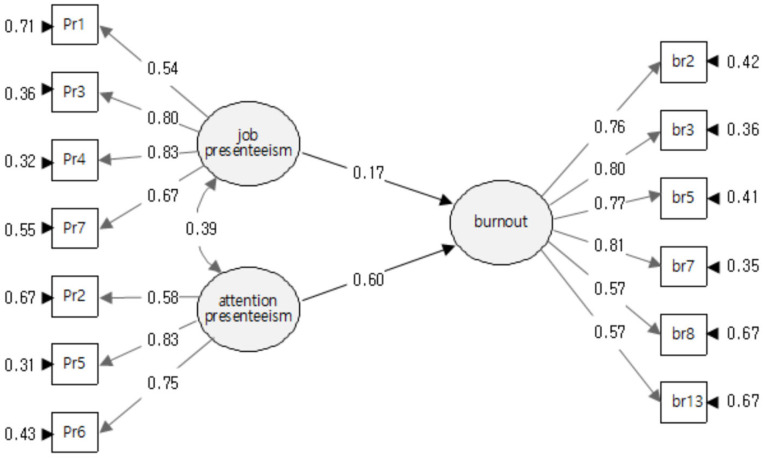
Structural equation modeling.

Regression analysis was conducted using a structural equation model to verify the effect of presenteeism among RMWs on burnout. The results are presented in Table 4 and Figure 2.

First, the results of analyzing the relationship between burnout and JP, which implies job impairment, showed a path regression coefficient estimate of 0.283 and a *z*-value of 3.390 with a significance probability of *p* < 0.001. This result indicates that JP had a significantly positive effect on burnout at the statistical significance level. Second, we found that AP, indicating impairment from attention distraction, showed an estimate of 0.851 and a *z*-value of 8.863 (*p* < 0.001). This also further indicates that AP also had a statistically significant positive effect on RMWs’ burnout.

Thus, we found that both JP and AP, derived from presenteeism, had very significant effects on RMWs’ burnout, and we also observed that AP (0.851) had a much higher effect than JP (0.283) on RMWs’ burnout in Korea.

### 3.4. Multiple Mediation Analysis

**Figure 3:**
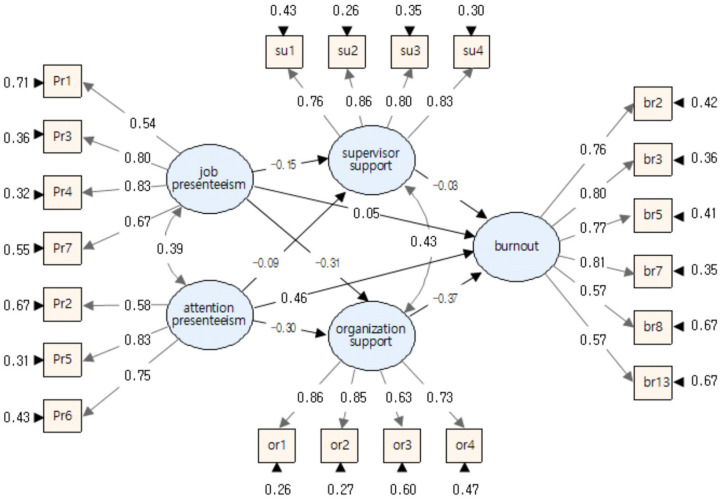
Multiple mediating effect verification model. (Note: standardized coefficients are reported).

To analyze the multiple mediating effects of OS and SS on the relationship between JP, AP, and burnout among RMWs, we conducted an analysis using the JSAP program with 5000 times of bootstrapping iterations at a 95% confidence interval (CI) (refer to Table 5 and Figure 3).

First, the results of the analysis using the bootstrapping technique for the multiple mediating model showed that the total mediating effect on the relationship between JP and burnout among RMWs was 0.272 (*p* < 0.001, CI = [0.153, 0.385]), confirming a significant mediating effect. In this relationship, the direct effect was 0.140 (CI = [0.022, 0.253]) and the indirect effect was 0.132 (CI = [0.079, 0.197]); both effects were found to be significant at the level of *p* < 0.05 and *p* < 0.001. Second, regarding the relationship between AP and burnout, the total mediating effect was 0.566 (*p* < 0.001, CI = [0.472, 0.660]), indicating that AP had a significant mediating effect on burnout. The direct effect of this relationship was 0.447 (CI = [0.353, 0.540]) and the indirect effect was 0.119 (CI = [0.078, 0.174]); both effects were significant at *p* < 0.001.

In the multiple mediating model, after inputting two mediating variables (OS and SS) into the relationship between JP, AP, and burnout among RMWs, the total mediating effect of AP on burnout was more than twice (0.294 = 0.566 − 0.272) that of JP on burnout. This indicates that impairment from attention distraction (AP) rather than job impairment (JP) due to presenteeism has a much greater effect on burnout among RMWs in Korea. These findings certify that the impairment from attention distraction (AP) due to presenteeism had a much stronger effect on burnout than the job impairment (JP) among Korean RMWs.

Additionally, the indirect mediating effect sizes between OS and SS on the paths of JP and burnout were 0.101 (*p* < 0.001, CI = [0.058, 0.159]) and 0.031 (*p* < 0.05, CI = [0.002, 0.073]), respectively. This indicates that the mediating effect of OS was almost three times greater than that of SS on the path between JP and burnout among RMWs. For the path between AP and burnout, the indirect mediating effects of OS and SS were 0.096 (*p* < 0.001, CI = [0.060, 0.147]) and 0.023 (*p* < 0.05, CI = [0.002, 0.053]), respectively. This also indicates that the mediating effect of OS was more than four times greater than that of SS. These two findings attest that OS plays a much better role than SS in mitigating the positive relationship between presenteeism and burnout among RMWs in Korea.

## 4. Discussion

This study investigated the impact of presenteeism among RMWs on burnout and verified the multiple mediating effects of OS and SS on this relationship. The major study findings are as follows.

First, by examining the relationship between the participants’ general characteristics and burnout, we found statistically significant differences in sex, marital status, and company type. Regarding sex, the number of female RMWs (3.42) was significantly higher than that of male workers (3.02). This finding was corroborated by a similar conclusion in Lee and Ma’s study [56], analyzing the relationship between sex and burnout among occupational therapists in Korea. It seems necessary to prepare policy alternatives to resolve these significant gender differences.

The analysis of differences in burnout based on marital status revealed higher burnout among unmarried participants than among married participants. Similar results were found in a meta-analysis of the factors affecting burnout among occupational therapists [57] and in a burnout study conducted on Spanish occupational therapists [58]. Having a spouse or family has previously been identified as a potentially stressful factor [59]. However, unlike unmarried participants, who are very likely to experience burnout, as reported by Maslach and Jackson [60], married participants are generally older and psychologically mature because they have more experience with emotional conflict and receive greater emotional support from their families. Therefore, a system involving support from colleagues, supervisors, or organizations is required to reduce burnout among unmarried individuals.

Analysis of differences in burnout based on company type showed the worst burnout among workers in private rehabilitation institutions (3.47), followed by nursing hospitals (3.40), public rehabilitation institutions (3.28), and general hospitals (3.12). This requires follow-up research on working conditions depending on the workplace type.

Second, we conducted a regression analysis using a structural equation model to verify the effects of presenteeism (JP and AP) on burnout among RMWs. Results showed that both factors significantly affected burnout. Particularly, it was found that loss of attention due to presenteeism had a greater effect on burnout than loss of job performance. This is meaningful as it provides empirical evidence that the effect of AP is greater than that of JP between the two sub-factors of presenteeism, affecting burnout among RMWs. Given that RMWs cannot focus on their work or on patients due to their lack of attention, management will need to actively intervene to eradicate presenteeism, which can lead to poor quality of medical services, medical malpractice, and a risk of wounds in workers themselves.

Third, as a result of analyzing the multiple mediating effects of OS and SS on the relationship between JP, AP, and burnout among RMWs, we found that both direct and indirect effects were significant at the *p* < 0.05 level. The significant influences of OS and SS have been verified as mediating factors that prevent or reduce burnout caused by presenteeism. This provides meaningful practical implications for improving RMWs’ work environment. Moreover, the absolute effect on burnout was found to have the greatest effect on AP. Regarding the mitigation effect of OS and SS, the fact that OS was far more effective than SS in the process of affecting burnout by both support types implies that workers are more affected by support from the organizational system than by personal support from their bosses. Management in medical rehabilitation institutions must consider these implications.

## 5. Conclusions

This study verified the significant discriminatory impacts of JP and AP as the two major impairment sub-factors derived from presenteeism on burnout among Korean RMWs (occupational therapists and physical therapists). Additionally, this study identified the positive mediating effects of organizational support and supervisory support as variables that could alleviate the impact of burnout and the relationship between them. This study also determined the differential roles of organizational support and supervisory support perceived by Korean RMWs in the positive relationship between presenteeism and burnout as a means of mitigating burnout syndrome.

Concurrently, the results of this study have certain implications. In terms of theoretical perspectives, the present study contributes to the literature on burnout by examining the relationship between presenteeism and burnout and by extending the current understanding of burnout and presenteeism to RMWs. First, our research model makes a valuable contribution to the literature by examining the causal relationship between presenteeism and burnout, in particular, with two key factors of presenteeism. Second, the current study provided the first empirical evidence of the discriminatory impacts between JP and AP in presenteeism on burnout. Third, the study identified the positive mediating effects of organizational support and supervisory support as alleviating variables against the impact of burnout. As for the practical perspectives, it is important to understand that the effect of AP was greater than that of JP for the two key sub-factors of presenteeism affecting burnout among RMWs. Management needs to pay more attention to finding ways of helping RMWs handle their stress, finish work, and allow time off from work rather than demanding RMWs to achieve their goals or solve their problems. And, as we realized that workers are more affected by support from the organizational system than by personal support from their boss, organizations planning to develop HR programs and policies can make the most of this finding to minimize burnout among RMWs.

Since this study limited RMWs only to occupational and physical therapists, there is a limitation in generalizing the findings. Therefore, follow-up studies are needed for all RMWs, including doctors, nurses, and social welfare workers, in the near future.

## Figures and Tables

**Table 1:** Differences in socio-demographics on burnout.

			Burnout			
			Mean	SD	F(t)	*p*
Gender	Male	148	3.02	0.851	−4.95	<0.001 ***
	Female	346	3.42	0.791		
Age	20 s	351	3.31	0.823	0.623	0.539
	30 s	104	3.29	0.884		
	Over 40	39	3.18	0.731		
Education	College	124	3.33	0.824	0.091	0.913
	University	318	3.29	0.819		
	Grad. School	52	3.28	0.913		
Job	OT ^(1)^	257	3.26	0.832	−0.941	0.348
	PT ^(2)^	237	3.33	0.826		
Marriage	Married	123	3.14	0.825	−2.38	0.018 *
	Unmarried	371	3.35	0.825		
Double Income	Yes	120	3.21	0.821	−1.35	0.179
	No	374	3.33	0.830		
Preschooler	No	432	3.33	0.821	1.92	0.056
	Yes	62	3.11	0.861		
Workplace	Gen. Hosp. ^(3)^	132	3.12	0.849	5.67	<0.001 ***
	Priv. Rehab. ^(4)^	163	3.47	0.783		
	Pub. Rehab. ^(5)^	87	3.28	0.812		
	Nursing Hosp. ^(6)^	90	3.40	0.809		
	Others	22	2.78	0.828		

* *p* < 0.05, *** *p* < 0.001; ^(1)^ OT: occupational therapist, ^(2)^ PT: physical therapist, ^(3)^ general hospital, ^(4)^ private rehabilitation hospital, ^(5)^ public rehabilitation hospital, ^(6)^ nursing hospital.

**Table 2:** Structural equation model fit index.

Index	Measurements	Results
Chi-square test (χ^2^, *p* > 0.05)	212.228 (df = 62, AIC = 16,152.8)	*p* < 0.01
Root mean square residual (RMR)	0.051	<0.08
Standardized root mean square residual (SRMR)	0.046	<0.05
Root mean square error of approximation (RMSEA)	0.059	<0.08
Comparative fit index (CFI)	0.943	>0.9
Normed fit index (NFI)	0.922	>0.9
Non-normed fit index (NNFI)	0.929	>0.9
Tucker–Lewis index (TLI)	0.930	>0.9
Bollen’s incremental fit index (IFI)	0.944	>0.9
Goodness of fit index (GFI)	0.987	>0.9

**Table 3:** Validity and reliability of latent variables.

Variable	JP	AP	OS	SS	Burnout
A.V.E.	0.518	0.528	0.519	0.598	0.663
Cronbach’s α	0.802	0.757	0.857	0.846	0.886

**Table 4:** Regressions of latent variables.

Dependent Variable	Latent Variable	Estimate ^(1)^	S.E.	*z*-Value	*p* (>|z|)	Std.lv ^(2)^	Std.All
Burnout	JP	0.283	0.083	3.390	0.001 ***	0.170	0.170
	AP	0.851	0.096	8.863	0.001 ***	0.609	0.609

*** *p* < 0.001; ^(1)^ unstandardized coefficients, ^(2)^ standardized coefficients.

**Table 5:** Multiple mediating effect verification.

Path		Effect	B ^(1)^	SE	*p*	95% CI	
						Lower	Upper
JP → burnout		Direct	0.140	0.049	0.004 **	0.022	0.253
		Indirect	0.132	0.025	0.001 ***	0.079	0.197
		Total	0.272	0.051	0.001 ***	0.153	0.385
AP → burnout		Direct	0.447	0.042	0.001 ***	0.353	0.540
		Indirect	0.119	0.021	0.001 ***	0.078	0.174
		Total	0.566	0.044	0.001 ***	0.472	0.660
JP	OS ^(2)^ → burnout	Indirect	0.101	0.023	0.001 ***	0.058	0.159
	SS ^(3)^ → burnout		0.031	0.015	0.038 *	0.002	0.073
AP	OS → burnout	Indirect	0.096	0.020	0.001 ***	0.060	0.147
	SS → burnout		0.023	0.011	0.043 *	0.002	0.053
OS → SS		Residual covariances	0.455	0.043	0.001 ***	0.359	0.567

* *p* < 0.05, ** *p* < 0.01, *** *p* < 0.001; ^(1)^ unstandardized coefficients, ^(2)^ OS: organizational support, ^(3)^ SS: supervisory support.

## Data Availability

The data presented in this study are available on request from the corresponding author.

## References

[1] Huang C., Wang Y., Li X., Ren L., Zhao J., Hu Y., Zhang L., Fan G., Xu J., Gu X. (2020). Clinical features of patients infected with 2019 novel coronavirus in Wuhan, China. Lancet.

[2] Ruskin K.J., Ruskin A.C., Musselman B.T., Harvey J.R., Nesthus T.E., O’Connor M. (2021). COVID-19, Personal Protective Equipment, and Human Performance. Anesthesiology.

[3] Loibner M., Hagauer S., Schwantzer G., Berghold A., Zatloukal K. (2019). Limiting factors for wearing personal protective equipment (PPE) in a health care environment evaluated in a randomised study. PLoS ONE.

[4] Wheeler H.H. (1997). A review of nurse occupational stress research. Br. J. Nurs..

[5] Elshaer N.S.M., Moustafa M.S.A., Aiad M.W., Ramadan M.I.E. (2019). Job Stress and Burnout Syndrome among Critical Care Healthcare Workers. Alex. J. Med..

[6] Ghahramani S., Lankarani K.B., Yousefi M., Heydari K., Shahabi S., Azmand S. (2021). A Systematic Review and Meta-Analysis of Burnout Among Healthcare Workers During COVID-19. Front. Psychiatry.

[7] Maslach C., Schaufeli W.B., Leiter M.P. (2001). Job Burnout. Annu. Rev. Psychol..

[8] Azoulay E., Cariou A., Bruneel F., Demoule A., Kouatchet A., Reuter D., Souppart V., Combes A., Klouche K., Argaud L. (2020). Symptoms of anxiety, depression, and peritraumatic dissociation in critical care clinicians managing patients with COVID-19. A cross-sectional study. Am. J. Respir. Crit. Care Med..

[9] Carmassi C., Foghi C., Dell’Oste V., Cordone A., Bertelloni C.A., Bui E., Dell’Osso L. (2020). PTSD symptoms in healthcare workers facing the three coronavirus outbreaks: What can we expect after the COVID-19 pandemic. Psychiatry Res..

[10] De Pablo G.S., Vaquerizo-Serrano J., Catalan A., Arango C., Moreno C., Ferre F., Shin J.I., Sullivan S., Brondino N., Solmi M. (2020). Impact of coronavirus syndromes on physical and mental health of health care workers: Systematic review and meta-analysis. J. Affect. Disord..

[11] Shanafelt T.D., Bradley K.A., Wipf J.E., Back A.L. (2002). Burnout and self-reported patient care in an internal medicine residency program. Ann. Intern. Med..

[12] Maslach C., Leiter M., Cooper C.L., Quick J.C. (2017). Understanding burnout. The Handbook of Stress and Health.

[13] Stehman C.R., Testo Z., Gershaw R.S., Kellogg A.R. (2019). Burnout, drop out, suicide: Physician loss in emergency medicine, part I. West. J. Emerg. Med..

[14] Aronsson G., Gustafsson K., Dallner M. (2000). Sick but yet at work. an empirical study of sickness presenteeism. J. Epidemiol. Community Health.

[15] Johns G. (2010). Presenteeism in the workplace: A review and research agenda. J. Organ. Behav..

[16] Böckerman P., Laukkanen E. (2009). Presenteeism in Finland: Determinants by gender and the sector of economy. Ege Acad. Rev..

[17] Baeriswyl S., Krause A., Elfering A., Berset M. (2016). How workload and coworker support relate to emotional exhaustion: The mediating role of sickness presenteeism. Int. J. Stress. Manag..

[18] Lazarus R.S., Folkman S. (1984). Stress, Appraisal and Coping.

[19] Demerouti E., Bakker A.B., Nachreiner F., Schaufeli W.B. (2001). The job demands-resources model of burnout. J. Appl. Psychol..

[20] Yang T., Zhu M., Xie X. (2016). The determinants of presenteeism: A comprehensive investigation of stress-related factors at work, health, and individual factors among the aging workforce. J. Occup. Health.

[21] Report A. Embracing the Age of Ambiguity Re-Invigorating the Workforce in a Rapidly Evolving World. https://www.aviva.co.uk/business/age-of-ambiguity/embracing-the-age-of-ambiguity/.

[22] Pei P., Lin G., Li G., Zhu Y., Xi X. (2020). The association between doctors’ presenteeism and job burnout: A cross-sectional survey study in China. BMC Health Serv. Res..

[23] Gillet N., Huyghebaert-Zouaghi T., Reveillere C., Colombat P., Fouquereau E. (2020). The effects of job demands on nurses’ burnout and presenteeism through sleep quality and relaxation. J. Clin. Nurs..

[24] Nwosu A.D., Ossai E., Onwuasoigwe O., Ezeigweneme M., Okpamen J. (2021). Burnout and presenteeism among healthcare workers in Nigeria: Implications for patient care, occupational health and workforce productivity. J. Public Health Res..

[25] Jalili M., Niroomand M., Hadavand F., Zeinali K., Fotouhi A. (2021). Burnout among healthcare professionals during COVID-19 pandemic: A cross-sectional study. Int. Arch. Occup. Env. Health.

[26] Demerouti E., Le Blanc P.M., Bakker A.B., Schaufeli W.B., Hox J. (2009). Present but sick: A three-wave study on job demands, presenteeism and burnout. Career Dev. Int..

[27] Wright T.A., Cropanzano R. (1998). Emotional exhaustion as a predictor of job performance and voluntary turnover. J. Appl. Psychol..

[28] Hobfoll S.E. (1988). The Ecology of Stress.

[29] Hobfoll S.E. (2001). The influence of culture, community, and the nested-self in the stress process: Advancing conservation of resources theory. Appl. Psychol. Int. Rev..

[30] Meijman T.F., Mulder G., Drenth P.J.D., Thierry H. (1998). Psychological aspects of workload. Handbook of Work and Organizational Psychology.

[31] Turpin R.S., Ozminkowski R.J., Sharda C.E., Collins J.J., Berger M.L., Billotti G.M., Baase C.M., Olson M.J., Nicholson S. (2004). Reliability and validity of the Stanford Presenteeism Scale. J. Occup. Environ. Med..

[32] West C.P., Dyrbye L.N., Shanafelt T.D. (2018). Physician burnout: Contributors, consequences and solutions. J. Intern. Med..

[33] Shanafelt T., Ripp J., Trockel M. (2020). Understanding and Addressing Sources of Anxiety Among Health Care Professionals During the COVID-19 Pandemic. JAMA.

[34] Shin J., McCarthy M., Schmidt C., Zellner J., Ellerman K., Britton M. (2022). Prevalence and predictors of burnout among occupational therapy practitioners in the United States. Am. J. Occup. Ther..

[35] Bakker A.B., Demerouti E., Taris T.W., Schaufeli W.B., Schreurs P.J. (2003). A multigroup analysis of the job demands-resources model in four home care organizations. Int. J. Stress Manag..

[36] Väänänen A., Toppinen-Tanner S., Kalimo R., Mutanen P., Vahtera J., Peiró J.M. (2003). Job characteristics, physical and psychological symptoms, and social support as antecedents of sickness absence among men and women in the private industrial sector. Soc. Sci. Med..

[37] Burke M.J., Borucki C.C., Hurley A.E. (1992). Reconceptualizing psychological climate in a retail service environment: A multiple-stakeholder perspective. J. Appl. Psychol..

[38] Ross R.R., Altmaier E.M., Russell D.W. (1989). Job stress, social support, and burnout among counseling center staff. J. Couns. Psychol..

[39] Lee R.T., Ashforth B.E. (1996). A meta-analytic examination of the correlates of the three dimensions of job burnout. J. Appl. Psychol..

[40] Halbesleben J.R. (2006). Sources of social support and burnout: A meta-analytic test of the conservation of resources model. J. Appl. Psychol..

[41] Eisenberger R., Huntington R., Hutchison S., Sowa D. (1986). Perceived Organizational Support. J. Appl. Psychol..

[42] Dawley D.D., Andrews M.C., Bucklew N.S. (2008). Mentoring, supervisor support, and perceived organizational support: What matters most?. Leadersh. Organ. Dev. J..

[43] Cropanzano R., Howes J.C., Grandey A.A., Toth P. (1997). The relationship of organizational politics and support to work behaviors, attitudes, and stress. J. Organ. Behav..

[44] Jawahar I.M., Stone T.H., Kisamore J.L. (2007). Role conflict and burnout: The direct and moderating effects of political skill and perceived organizational support on burnout dimensions. Int. J. Stress Manag..

[45] Brislin R.W. (1986). The Wording and Translation of Research Instruments.

[46] Maslach C., Jackson S.E. (1981). The measurement of experienced burnout. J. Occup. Behav..

[47] Yoon J., Lim J. (1999). Organizational support in the workplace: The case of Korean Hospital Employees. Hum. Relat..

[48] Stevens J. (2002). Applied Multivariate Statistics for the Social Sciences.

[49] Moss S. Fit Indices for Structural Equation Modeling. https://www.sicotests.com/newpsyarticle/Fit-indices-for-structural-equation-modeling/.

[50] Browne M.W., Cudeck R. (1992). Alternative ways of assessing model fit. Sociol. Methods Res..

[51] Hu L.-t., Bentler P.M. (1999). Cutoff criteria for fit indexes in covariance structure analysis: Conventional criteria versus new alternatives. Struct. Equ. Model. A Multidiscip. J..

[52] Byrne B.M. (1994). Structural Equation Modeling with EQS and EQS/Windows: Basic Concepts, Applications, and Programming.

[53] Schumacker E., Lomax G. (2016). A Beginner’s Guide to Structural Equation Modeling.

[54] Fornell C., Larcker D.F. (1981). Evaluating structural equation models with unobservable variables and measurement error. J. Mark. Res..

[55] Cronbach L.J. (1951). Coefficient alpha and the internal structure of tests. Psychometrika.

[56] Lee S.-I., Ma S.-R. (2018). A study on burnout, emotional labor, and self-efficacy of occupational therapists. J. Korea Entertain. Ind. Assoc..

[57] Park E.-Y. (2021). Meta-Analysis of Factors Associated with Occupational Therapist Burnout. Occup. Ther. Int..

[58] Escudero-Escudero A.C., Segura-Fragoso A., Cantero-Garlito P.A. (2020). Burnout Syndrome in Occupational Therapists in Spain: Prevalence and Risk Factors. Int. J. Environ. Res. Public Health.

[59] Poulsen A.A., Meredith P., Khan A., Henderson J., Castrisos V., Khan S.R. (2014). Burnout and work engagement in occupational therapists. Br. J. Occup. Ther..

[60] Maslach C., Jackson S.E. (1985). The role of sex and family variables in burnout. Sex Roles.

